# Bibliometric analysis of calcium channel research (2010–2019)

**DOI:** 10.1080/19336950.2020.1788903

**Published:** 2020-07-13

**Authors:** Jingjing Shi, Huan Wang, Shuqing Shi, Guozhen Yuan, QiuLei Jia, Shuai Shi, Xuesong Zhang, Yuanhui Hu

**Affiliations:** a Guanganmen Hospital, China Academy of Chinese Medical Sciences, Beijing, China; b Graduate School of Beijing University of Chinese Medicine, Beijing, China

**Keywords:** Bibliometric, calcium channel, citespace, visual analysis

## Abstract

Calcium channels are involved in pathologies across all the major therapeutic areas involving the cardiac, neurological, metabolic, and respiratory systems. Although calcium channels have been the hotspot of multidisciplinary research for decades, the hotspots and frontier trends of calcium channel research have not been comprehensively analyzed by bibliometrics. Here, we collected scientific publications on calcium channel research in the past decade to explore the hotspots and frontier directions of calcium channel research by bibliometric analysis. Publications were retrieved from the Web of Science Core Collection (WOSCC) database from 2010 to 2019. Citespace5.6 R5 was used to perform bibliometric analysis on the countries, institutions, authors, and related research areas. In total, 26,664 articles were analyzed. The United States and the University of California are the most productive country and institution for calcium channel research. The most productive researchers were Lang, Florian, Zamponi, Gerald W, and Jan, Chung-Ren. PLoS One had the most significant number of publications (986). Research hotspots can be summarized as the regulation mechanism of calcium channels, calcium channel blockers, and ryanodine receptor. The research frontiers were the effect of calcium channel on cell proliferation, gene mutation, calcium channels in neuropathic pain, and calcium-signaling pathway. This is the first report to visualize and analyze hotspots and emerging trends in calcium channel research.

Calcium channels are protein complexes that present on the cell membrane, endoplasmic reticulum or sarcoplasmic reticulum and allow calcium ions to flow through biological membranes quickly and selectively. Activation of calcium channels can promote the increase of free calcium ion concentration in cytoplasm. Calcium ions (Ca ^2+^) impact nearly every aspect of cellular life [1], including muscular contraction, neurotransmitter release, hormone secretion, and cell excitability control, cell cycle regulation, and cell communication, gene expression, cell metabolism, germ cell maturation, and fertilization, etc., [2,3].

According to different classification methods, calcium ion channels can be divided into the following categories: 1.Voltage-operated channels (VOCs); 2. Receptor-operated channels (ROCs); 3. Second-messenger-operated channels (SMOCs); 4. Transient receptor potential (TRP) ion-channels; 5. Inositol 1,4,5-trisphophate receptors (IP_3_Rs); 6. Ryanodine receptors (RYRs) [4].

Disturbance of calcium channels affects many diseases. Disorders of calcium channel function are associated with various cardiovascular diseases, such as atrial fibrillation, hypertension, and long QT syndrome [5]. Calcium is a ubiquitous mediator of many cell functions in the central nervous system (CNS) and has a pleiotropic effect in Alzheimer’s disease, Parkinson’s disease, Huntington’s disease, neuropsychiatric diseases, and other CNS disorders [6]. Studies have shown that calcium channel blockers have beneficial effects on obesity-related metabolic pathology [7]. And stable breathing requires the excitatory synaptic transmission of calcium channels [8].

Calcium channels are involved in pathologies across all the major therapeutic areas involving the cardiac, neurological, metabolic, and respiratory systems. Research on drugs acting on different calcium channel subtypes or subunits may have broad prospects in future therapeutic interventions [9]. Therefore, the use of bibliometric methods to sort out and summarize the existing research on calcium channel may provide better information for the study of calcium channels.

A bibliometric study can calculate the productivity of institutions, countries, and authors and explore research hotspots/frontiers in specific fields [10,11]. CiteSpace is one of the bibliometric visualization tools for data analysis and visualization [12], and it can visualize and analyze emerging trends and transition patterns in the scientific literature.

Although calcium channels have been the hotspot of multidisciplinary research for decades, no bibliometric studies regarding the trends in calcium channel research activity over the past decade have been published. Here, we collected scientific publications on calcium channel research in the past decade, using bibliometrics and visual analysis to explore the hotspots and frontier directions of calcium channel research and hope to provide researchers with some useful guidance.

## Data collection

The data search was conducted on 22 April 2020. The retrieved data were collected within 1 day to avoid any potential deviation due to the daily updating of the database. The search keywords entered into the database were as follows: TS = (calcium channel* OR calcium ion channel* OR VGCC* OR VDCC*) and language:(English). The data for analysis were retrieved from the Science Citation Index Expanded (SCI-expanded) of Web of Science Core Collection (WoSCC) database from 2010 to 2019.

In this study, the data were downloaded directly from the database as secondary data without further animal experiments. Therefore, no moral approval is required.

**Figure 1.**
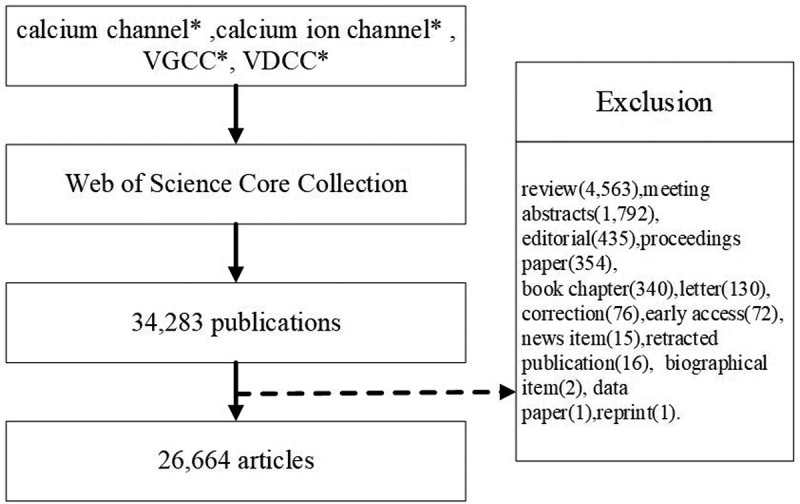
Flow chart of calcium channel researches inclusion.

In total, 34,283 publications were obtained, and the following document were excluded: review(4,563), meeting abstracts(1,792), editorial(435), proceedings paper(354), book chapter(340), letter(130), correction(76), early access(72), news item(15), retracted publication(16), biographical item(2), data paper(1) and reprint(1). In total, 26,664 articles were analyzed. The retrieval strategy of the experiments is shown in Figure 1.

## General information and annual publication output

**Figure 2.**
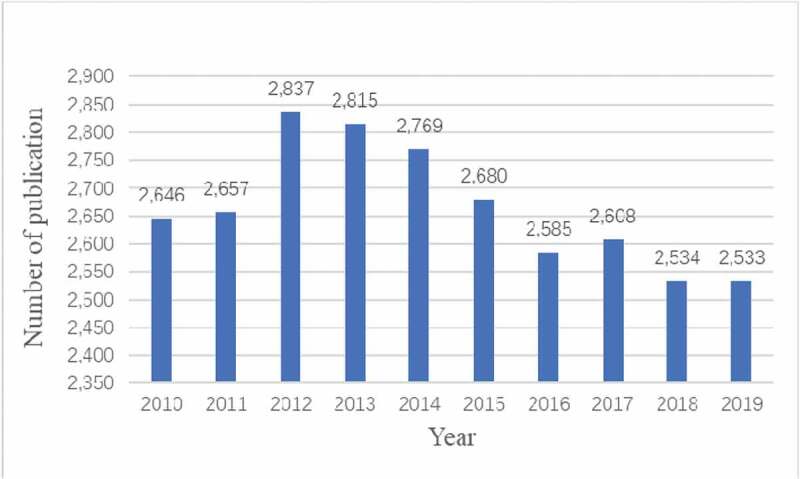
The number of annual publications on calcium channel research from 2010 to 2019.

A total of 26,664 articles were retrieved. To explore the trends of calcium channel research, we showed the number of articles per year in the form of a histogram. As shown in Figure 2, the number of publications on calcium channel research remained stable high in the past 10 years, the average annual number of publications was 2,666. It explained that the research on calcium channels was a popular research direction. The annual number of articles published in 2012–2014 was more than 2,700, which was the rapid development period of calcium channel research. The number of articles published in 2015–2019 declined, but it has remained at more than 2,500. The results showed that the field of calcium channel research had evolved from rapid to stable development in the past decade.

## Active countries and institutions

**Figure 3.**
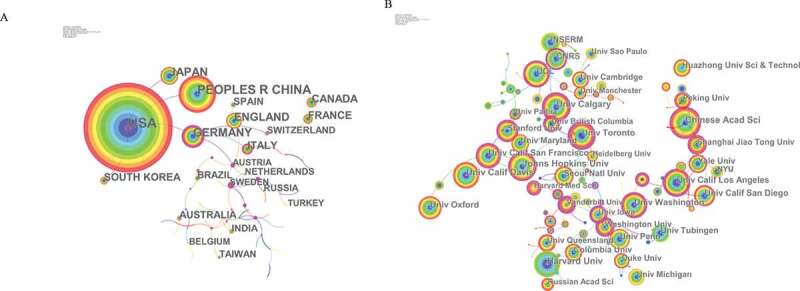
The analysis of countries and institutions. (a). Network of countries/territories engaged in calcium channel research; (b). Network of institutions engaged in calcium channel research.

Co-occurrence map provides valuable information and helps researchers to identify the cooperative relationship [13]. Table 1 lists the top 10 countries and institutions contributed to publications on calcium channel. Countries and institutions co-occurrence maps are shown in Figure 3(a,b).

**Table 1.** The top 10 countries and institutions contributed to publications on calcium channel research.

Rank	Country/Territory	Frequency	Institution	Frequency
1	USA	9,781	University of California	1,143
2	Peoples R China	4,276	Institut National de la Sante et de la Recherche Medicale (INSERM)	693
3	Germany	2,388	Harvard University	620
4	Japan	2,197	University of Texas System	524
5	England	1,921	University of London	477
6	Canada	1,412	National Institutes of Health (NIH)	465
7	France	1,273	Pennsylvania Commonwealth System of Higher Education Pcshe	461
8	Italy	1,238	Chinese Academy of Sciences	375
9	South Korea	1,013	Johns Hopkins University	336
10	Spain	794	University College London	315

Researchers from more than 132 countries/territories contributed to the 26,664 articles on calcium channel research. The USA, Peoples R China, Germany, Japan, and England were the top 5 productive countries (Table 1). The United States published the most papers (9,781 articles), followed by China (4,276 articles), and they were the two critical countries in calcium channel research. Figure 3(a) shows that the United States attached great importance to cooperation, and had close collaborations with China, Germany, Japan, and South Africa. Figure 3(b) shows that American institutions published most of the publications. The University of California produced the highest number of publications on calcium channels (1,143), followed by Institut National de la Sante et de la Recherche Medicale (INSERM) (693) and Harvard University (630). Co-occurrence map of institutions showed that scientific cooperation among institutions was greatly affected by the geographical location, and there are more cooperations among institutions in the same region.

## Active journals

The 26,664 articles were published in 2,999 journals, 10 journals have published more than 250 articles in the calcium channel field. Table 2 lists the top 10 journals that published articles on calcium channel research. Plos One had the highest number at 986 (3.70%) (IF_2019_ = 2.776), followed by the journal of biological chemistry published 646 papers (2.42%) (IF_2019_ = 4.106) and the journal of neuroscience ranked third at 559 papers (2.10%) (IF_2019_ = 6.074).

**Table 2.** The Top 10 journals that published articles on calcium channel research.

Rank	Journal	Frequency (%) N = 2,999	IF 2019	Country Affiliation
1	PLoS One	986(3.70%)	2.776	United State
2	The journal of biological chemistry	646(2.42%)	4.106	United State
3	Journal of neuroscience	559(2.10%)	6.074	United State
4	Scientific reports	468(1.76%)	4.011	United State
5	Proceedings of the National Academy of Sciences of the United States of America	466(1.75%)	9.580	England
6	Journal of Physiology-London	399(1.50%)	4.950	England
7	Cell Calcium	374(1.40%)	3.932	Netherlands
8	Biochemical and biophysical research communications	283(1.06%)	2.705	United States
9	European journal of pharmacology	274(1.03%)	3.170	United States
10	American journal of physiology.Cell physiology	264(0.99%)	3.553	United States

## Active authors

Author co-occurrence map can provide information on influential research groups and potential collaborators. It can help researchers to find potential collaborators [14].

**Figure 4.**
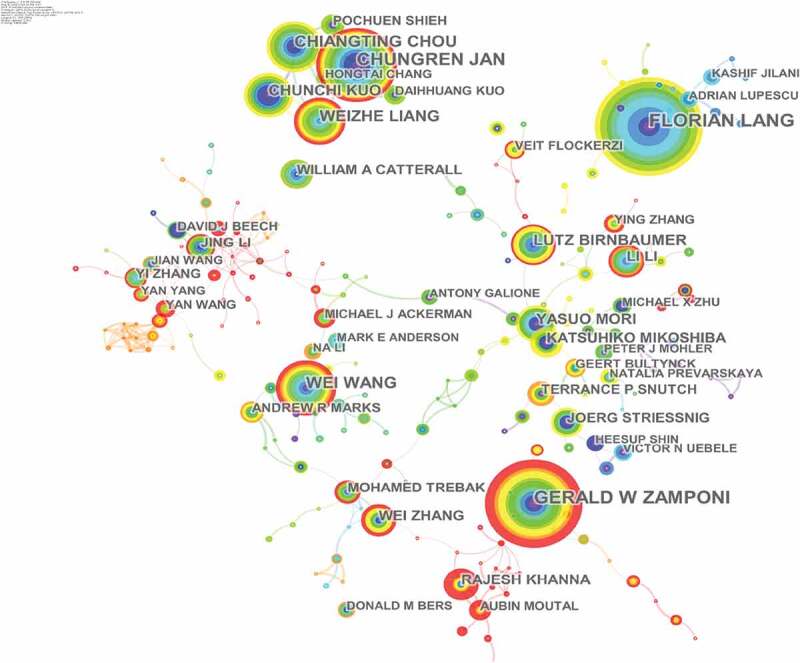
The network of authors contributed to calcium channel research.

More than 90,000 authors contributed 26,664 articles related to calcium channel research. Figure 4 shows the network of authors contributed to calcium channel research, and the top 10 active authors are listed in Table 3. In the network of authors contributed to calcium channel research, the largest node was Lang, Florian (93articles) who mainly focused on suicidal erythrocyte death [15,16] and regulation of STIM1/Orai1-dependent Ca^2+^ signaling [17,18]. Zamponi, Gerald W was the second highly published author. His research focused on the regulation of calcium channels [19,20] and their roles in the nervous system [21,22].

**Table 3.** The top10 active authors, in calcium channel research.

Rank	Author	Freq
1	Lang, Florian	93
2	Zamponi, Gerald W	84
3	Jan, Chung-Ren	71
4	Wei Wang	53
5	Naziroglu, Mustafa	51
6	Chou, Chiang-Ting	49
7	Liang, Wei-Zhe	46
8	Kuo, Chun-Chi	42
9	Birnbaumer, Lutz	39
10	Mori, Yasuo	35

## Co-cited references

**Figure 5.**
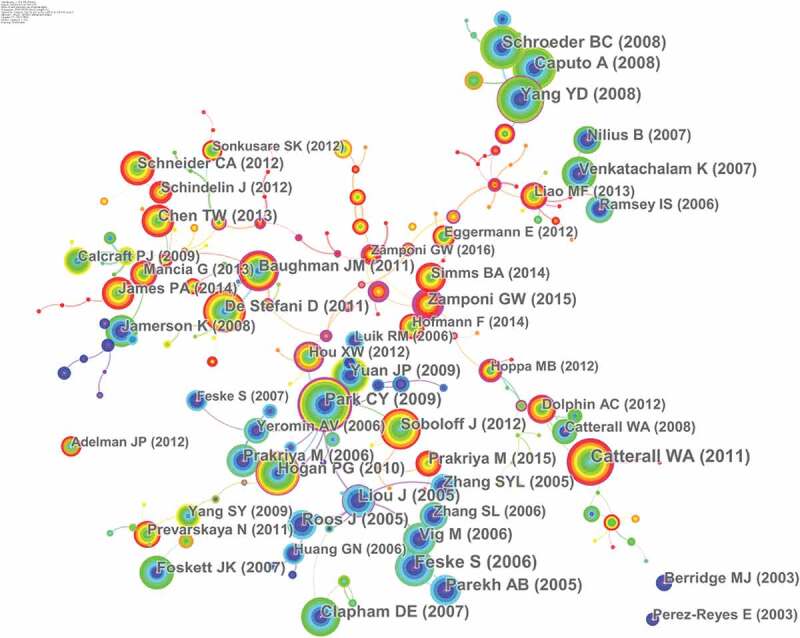
The analysis of Co-cited references: Co-citation network of references from publications on calcium channel research.

Twenty-six thousand six hundred sixty-four articles were visualized and analyzed using CiteSpace with a time span from 2010 to 2019, and a time slice of 1 was chosen for the analysis of the co-cited references. The network of co-cited references on calcium channels consists of references with higher centrality and citation counts which is presented in Figure 5. The highly cited references were analyzed to determine the key knowledge base in the field. The top 10 highest co-cited references are summarized in Table 4. Feske S, Liou J, Park CY, and Roos J mainly focused on STIM1 and CRACM1 (Orai1) regulating CRAC channels. Catterall WA, Parekh AB, and Clapham DE reviewed calcium channels and calcium signaling. Yang YD, Caputo A, and Schroeder BC mainly studied TMEM16A is a membrane protein associated with calcium-dependent chloride channel activity [23–25]. In2005, Roos J proposed that STIM1 played an essential role in Store-operated Ca^2+^ (SOC) channels [26]; Liou J’s study suggests that STIM proteins function as Ca^2+^ store sensors in the signaling pathway connecting Ca^2+^store depletion to Ca^2+^ influx [27]. In 2006 Feske S proposed that Orai1 is an essential component or regulator of the CRAC channel complex. Then in 2009, Park CY established the molecular mechanism for store-operated Ca^2+^ entry and found that the direct combination of STIM1 and Orai1 drives the accumulation and activation of CRAC channels at the endoplasmic reticulum – plasma membrane junctions. And thus these articles laid the foundation for the study of STIM1 and CRACM1 (Orai1) regulating CRAC channels. Clapham DE reviewed the principles of Ca^2+^ signal transduction in 2007 [1]. Parekh AB and Catterall WA reviewed store-operated calcium channels [28] and voltage-gated calcium channels [29]. These three reviews provided theoretical basis for the study of calcium channels. The highly co-cited references on TMEM16A were mainly published in 2008.

**Table 4.** The top10 Co-cited references (CR) in calcium channel research.

Rank	Freq	Author	Year	Source	Co-cited Reference
1	287	Feske, S	2006	Nature	A mutation in Orai1 causes immune deficiency by abrogating CRAC channel function
2	283	Catterall WA	2011	Cold Spring Harbor perspectives in biology	Voltage-gated calcium channels.
3	237	Liou J	2005	Current biology: CB	STIM is a Ca2+ sensor essential for Ca2+-store-depletion-triggered Ca2+ influx.
4	237	Yang YD	2008	nature	TMEM16A confers receptor-activated calcium-dependent chloride conductance.
5	233	Parekh AB	2005	Physiological reviews	Store-operated calcium channels.
6	230	Caputo A	2008	science	TMEM16A, a membrane protein associated with calcium-dependent chloride channel activity.
7	228	Schroeder BC	2008	cell	Expression cloning of TMEM16A as a calcium-activated chloride channel subunit.
8	214	Park CY	2009	cell	STIM1 clusters and activates CRAC channels via direct binding of a cytosolic domain to Orai1.
9	210	Roos J	2005	The Journal of cell biology	STIM1, an essential and conserved component of store-operated Ca2+ channel function.
10	209	Clapham DE	2007	cell	Calcium signaling.

## Research area analysis

**Figure 6.**
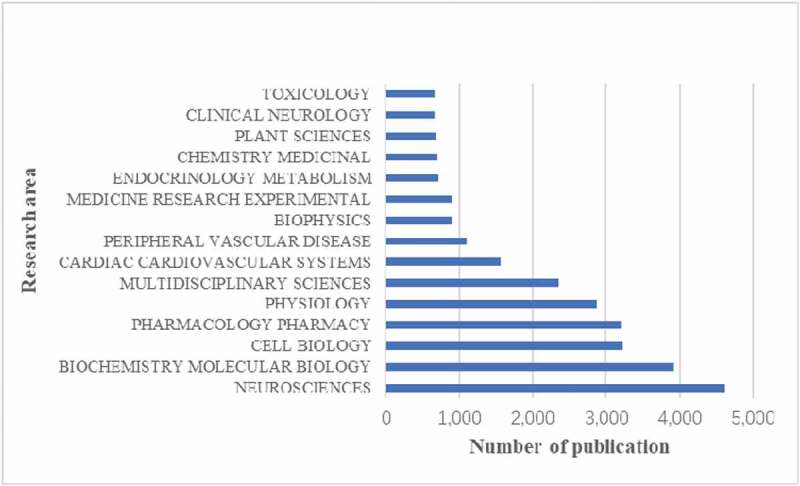
The 15 research areas on calcium channel research.

Figure 6 shows the top 15 research areas that appeared in publications related to calcium channel research from 2010 to 2019. Neurosciences, biochemistry and molecular biology, cell biology are the three areas where calcium channels are more studied.

## Keyword co-occurrence and burst

Keywords represent the main content of research. Keyword co-occurrence analysis provides a reasonable description of research hotspots, and burst keywords can represent research frontiers over a period of time [30].

**Figure 7.**
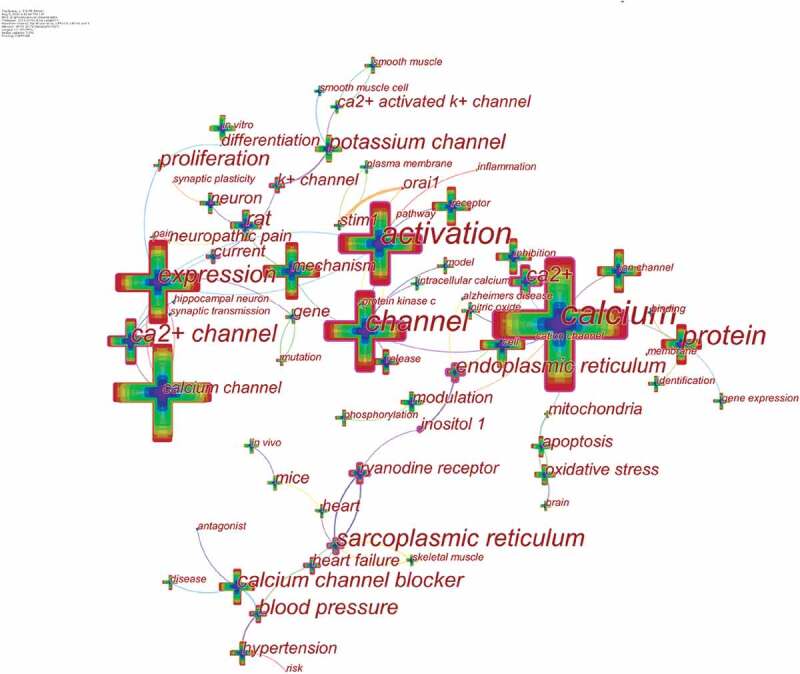
The analysis of keywords in calcium channel research.

CiteSpace 5.6.R5 were used to construct a knowledge map of keyword co-occurrence (Figure 7) and identified the top 20 keywords in calcium channel research articles from 2010 to 2019 (Table 5), according to frequency. The top keywords were “expression,” “activation,” “mechanism,” “cell,” “protein,” “receptor,” “calcium channel blocker,” “inhibition,” “rat,” “potassium channel,” “hypertension,” “in vitro,” “release,” “neuron,” “modulation,” “oxidative stress,” “apoptosis,” “nitric oxide,” “ryanodine receptor,” and “gene expression.”Therefore, research hotspots can be summarized in the following aspects:

**Table 5.** Top 20 keywords in terms of frequency in potassium channel research.

Rank	Keyword	Frequency	Rank	Keyword	Frequency
1	Expression	2865	11	Hypertension	1058
2	Activation	2792	12	In vitro	1021
3	Mechanism	1922	13	Release	1014
4	Cell	1606	14	neuron	995
5	Protein	1484	15	modulation	978
6	Receptor	1439	16	oxidative stress	948
7	Calcium channel blocker	1326	17	Apoptosis	885
8	Inhibition	1324	18	Nitric oxide	880
9	Rat	1199	19	Ryanodine receptor	815
10	Potassium channel	1058	20	Gene expression	805

### The regulation mechanism of calcium channels

As an important second messenger in cells, calcium ions are crucial in regulating various intracellular signaling pathways that control key cellular functions. Research on the regulation mechanism of calcium channels has always been a research hotspot, especially the research on [31] on STIM1 and CRACM1 (Orai1) regulating CRAC current. CRACM1(Orai1) is a pore subunit of the CRAC channel [32,33], and stromal interaction molecule 1 (STIM1) in the endoplasmic reticulum activates ORAI1-CRAC channels [34,35].

### Calcium channel blockers

Calcium channel blockers are a class of drugs that selectively block voltage-dependent calcium channels and inhibit the flow of Ca^2+^ into cells, and their development is rapid. Calcium channel blockers can be divided into dihydropyridines, phenylalkylamines, benzothiazepines, flunarizine, prenylamine based on the chemical structure and pharmacological characteristics.

### Ryanodine receptor

Ryanodine receptors (RyRs) are located in the sarcoplasmic/endoplasmic reticulum and are responsible for releasing Ca2+ from intracellular storage during excitation-contraction coupling, which is an important class of intracellular calcium release channel. RyRs are the largest known ion channels (>2MDa) and exist as three mammalian isoforms (RyR 1–3) [36].

Keywords were identified and analyzed using strong citation bursts (Table 6) to explore the frontiers of research. In Table 6, the red line indicates the time period during which the burst keyword appears [37]. As shown in Table 6, the keywords that had strong bursts after 2015 were “proliferation” (2015–2017), “mutation” (2016–2019), “neuropathic pain” (2017–2019), and “pathway” (2017–2019). The top four research frontiers of calcium channel research were as follows:

**Table 6.** Top 8 keywords with the strongest citation bursts.

Keywords	Year	Strength	Begin	End	2010–2019
Binding	2010	19.0215	**2010**	2011	
Smooth muscle cell	2010	41.2554	**2010**	2013	
Hippocampal neuron	2010	53.9931	**2010**	2011	
Protein kinase c	2010	60.3525	**2010**	2012	
Proliferation	2010	12.6853	**2015**	2017	
Mutation	2010	38.2867	**2016**	2019	
Neuropathic pain	2010	60.2029	**2017**	2019	
Pathway	2010	48.3937	**2017**	2019	

### Effect of calcium channel on cell proliferation

There is increasing evidence that calcium channels are involved in the occurrence and development of tumors. Calcium channels play essential roles in various forms of cancer. Disturbance of Ca^2+^ homeostasis leads to canceration or tumor cell proliferation [38].

### Gene mutation

Gene mutations encoding calcium channels lead to hereditary calcium channel disease. Studies have shown that CACNA1A gene mutations associated with some nervous system disease, such as familial hemiplegic migraine type 1, episodic ataxia type 2, and spinocerebellar ataxia type 6 [39]. Another study showed that mutations in the CACNA1 F gene could cause Aland Island eye disease [40].

### Calcium channels in neuropathic pain

Cav3.2 calcium channel plays a vital role in the nociceptive signal transduction of primary afferent pain pathway [41]. One study showed that dysregulation of USP5 SUMOization after peripheral nerve injury might be an essential cause of changes in Cav3.2 channel activity under neuropathic pain [42].

### Calcium signaling pathway

Changes in the primary and modulatory roles of calcium signaling are a contributory factor responsible for the onset of a large number human diseases [2]. The regulation mechanism of calcium-signaling pathway is still the frontier in calcium channel research.

## Conclusions

Based on the WOSCC database, bibliometric and Visual analysis were used to study the characteristics of calcium channel research results from 2010 to 2019. Over the past decade, the number of publications on calcium channel remained stable high. The three hot spots of calcium channel research were the regulation mechanism of calcium channels, calcium channel blockers, and ryanodine receptor. The top four research frontiers were the effect of calcium channel on cell proliferation, gene mutation, calcium channels in neuropathic pain, and calcium signaling pathway. Bibliometric analysis of the literature on the calcium channels was important in allowing researchers to identify cooperations, find research hotspots, and predict the frontiers of calcium channel research.
